# A Survey of Clinical Usage of Non-steroidal Intra-Articular Therapeutics by Equine Practitioners

**DOI:** 10.3389/fvets.2020.579967

**Published:** 2020-10-22

**Authors:** Ana Velloso Alvarez, Lindsey H. Boone, Amy Poulin Braim, Jenifer S. Taintor, Fred Caldwell, James C. Wright, Anne A. Wooldridge

**Affiliations:** ^1^Department of Clinical Sciences, Auburn University, Auburn, AL, United States; ^2^Zoetis, Parsippany, NJ, United States; ^3^Department of Pathobiology, Auburn University, Auburn, AL, United States

**Keywords:** survey, equine, sports medicine, non-steroidal therapeutics, joint disease, biological therapies, practitioners

## Abstract

There are several non-steroidal intra-articular therapeutics (NSIATs) available for use by equine practitioners for the treatment of performance-limiting joint-related pathology. Information is limited on perceived clinical efficacy, recommended treatment protocols, and associated complications. Our objective with this cross-sectional survey was to investigate the current clinical usage of NSIATs by equine practitioners. An electronic cross-sectional convenience survey inquiring about the use of steroidal and NSIATS (platelet-rich plasma, autologous conditioned serum, autologous protein solution, cellular therapies, and polyacrylamide hydrogel) was distributed internationally to equine practitioners. A total of 353 surveys were completed. NSIATs were used by 87.5% of the participants. Corticosteroids and hyaluronic acid remain the intra-articular therapeutic of choice among practitioners, followed by autologous conditioned serum, platelet-rich plasma and autologous conditioned protein. Polyacrylamide hydrogel was the least used. Practitioners were more likely to use NSIATs if their caseload was > 50% equine (*P* < 0.001), they treated more than 10 horses intra-articularly per month (*P* < 0.001), and horses treated were considered English sport horses (*P* = 0.02). Years in practice and practice location did not influence the use of NSIATs. One of the most common reasons why NSIATs were chosen was to treat acute articular pathologies. As survey limitations, answers to questions regarding clinical response and complication rates were based on subjective estimation and practitioners recall, not clinical records. In conclusion, corticosteroids remain the most widely used intra-articular therapeutic. Among the NSIATs, blood-based products are more commonly used by practitioners, followed by cellular and synthetic products. Equine practitioners frequently use NSIATs, choosing to treat acute joint pathology more than previously reported.

## Introduction

Within the equine industry, lameness as a result of musculoskeletal pain, particularly osteoarthritis (OA), has significant economic impact and is one of the top reasons for veterinary evaluation and treatment (1, 2). Several different intra-articular medications are available to equine practitioners to help alleviate musculoskeletal pain, especially when initial rest and systemic anti-inflammatory therapy is unsuccessful (3).

Currently, the mainstay of intra-articular therapy is modification of disease symptoms through transient reduction of inflammation via administration of corticosteroids with or without hyaluronic acid (4). Recently, non-steroidal intra-articular therapies (NSIATs), such as biological and synthetic products, have become more popular. NSIATs have been shown to possess limited disease-modifying properties, such as slowing down disease progression and enhancing the quality of repair tissue (5–11).

Equine practitioners have used NSIATs for years (12), and currently, there are many options available on the market. In the literature, there is limited information regarding the clinical experience of practitioners with these products, such as their product preference and treatment protocol (4, 12). This survey was not a hypothesis-driven study. The objectives of this study were: (1) to explore how equine practitioners use NSIATs, specifically autologous conditioned plasma (also known as platelet-rich plasma, PRP), autologous conditioned serum (ACS), autologous protein solution (APS), cellular products (i.e., stem/stromal/progenitor cell therapy), and polyacrylamide hydrogel, and (2) to observe if NSIATs are more frequently used by equine practitioners than previously reported in the literature. The survey would provide information as to which NSIATs are more commonly used by equine practitioners as well as subjective clinical efficacy, treatment protocols commonly employed, and complications associated with product use.

## Materials and Methods

The project was reviewed and approved by the Institutional Review Board for the Protection of Human Subjects in Research (18-486 EX 1811). An electronic questionnaire (Qualtrics XM software, Provo, Utah, USA) inquiring about the use of 5 different NSIATs was distributed internationally to equine practitioners between January 2019 and October 2019. The survey was distributed to an estimated 10,000 equine practitioners. The questionnaire link was distributed to members of the American Association of Equine Practitioners through the Spur of the Moment Newsletter. The link was also distributed by the European College of Veterinary Surgeons to their Diplomats. Equine practitioners at 26 of 30 USA veterinary schools were contacted by email addresses obtained through university websites. Through a collaboration with Zoetis, the survey link was also distributed to equine customers in the USA who purchased pain and sedation products commonly used for lameness workup, diagnosis, and treatment. Lastly, the questionnaire was distributed through equine practitioner groups on social media (Facebook groups: Equine Vet-2-Vet and Equine lameness vets). Each participant was given a unique identifier based on email and IP address, to avoid duplication of answers. The survey additionally collected demographic information about each practitioner's practice and experience (geographic location, primary equine discipline treated, years in practice, frequency of intra-articular injection).

The survey contained a total 59 questions, with a combination of multiple-choice and rank questions, inquiring about PRP, ACS, APS, cellular therapies and polyacrylamide hydrogel. Within the survey, brief descriptions of each NSIAT were provided before specific questions (Table 1). If the practitioner did not use a product, product questions were eliminated from the survey. Questions regarding the use of NSIATs included: rank and justification of product preference, clinical usage, subjective assessment of clinical efficacy, treatment protocol used, and frequency of an observed inflammatory response (joint flare) after intra-articular administration. A copy of the survey has been provided, Supplementary Item 1. Before distributing the survey to the public, it was tested and evaluated by 10 individuals that did not include the investigators. Answers from these individuals were not included in the results.

**Table 1:** Brief description of each NSIATs provided to practitioners prior to questioning.

**NSIAT**	**NSIAT Description**
*Autologous conditioned plasma (ACP)*	PRP is a product obtained from the horse's blood. The blood is filtered or centrifuged to obtain plasma with an increased number of platelets rich in growth factors.
*Autologous conditioned serum (ACS)*	ACS, also known as IRAP (Orthokine® vet irap 10 or 60, IRAP II^TM^ System), is obtained from the horses' blood following collection into specialized syringes and whole blood incubation. The serum is then collected and administered, or aliquots are frozen for subsequent injection.
*Autologous protein solution (APS):*	APS (i.e., Pro-Stride™ APS) is an autologous product obtained from the horse's blood. The blood is first processed using a kit and centrifugation to obtain plasma with concentrated platelets. This plasma is then harvested and processed in a kit that allows exposure of the cellular components of the plasma to polyacrylamide beads, enhancing their production of anti-inflammatory proteins during a second centrifugation cycle.
*Cellular therapeutics*	Cellular therapeutics would include the following products: • Cells (stem/stromal and/or progenitor) contained within **tissue particles** (i.e., Pulpcyte^®^ Vet Graft). These products are typically shipped directly from the company. • Progenitor and stem/stromal cell **concentrates**. These products are obtained after harvesting tissue (adipose or bone marrow) and concentration of the cells from the tissue via centrifugation with or without prior tissue digestion (i.e., Adipose-derived stromal vascular fraction or bone marrow aspirate concentrate). • **Cultured** cellular therapy. These products are obtained after harvesting tissue (adipose, bone marrow, blood, etc.) and sending the tissues to a commercial laboratory for culture. The cultured cells would then be shipped back to the practitioner for injection at least 2 weeks or more after the tissue harvest.
Polyacrylamide hydrogel	Polyacrylamide Hydrogel (PAHG, Noltrex^TM^ Vet, or Arthramid®Vet, Aquamid^®^) is a synthetic product injected intra-articularly. It is incorporated into the synovial lining and provides enhanced viscoelasticity to the synovial fluid.

### Statistical Analysis

Survey data were summarized and reported using percentages and/or rankings. Chi-Square analyses (Qualtrix XM software, Provo, Utah, USA) were conducted to evaluate the influence of practitioner geographic location (USA vs. non-USA), years of experience, lameness caseload, number of horses injected intra-articularly on a monthly and yearly basis, and primary discipline treated. Effect of allogenic or autologous cell therapy on flare rate and the practitioner's geographic location (USA vs. non-USA) with use of polyacrylamide hydrogel was evaluated using a chi-square analysis as well. Significance was set at *P* < 0.05. For questions in which practitioners were asked to rank responses, the response with the lowest average number (ranked as 1) was reported as the preferred choice, followed in descending order to the least preferred response. Participants were required to rank at least 3 options, the median and the interquartile range were calculated for each NSIAT and reported.

## Results

A total of 473 equine practitioners participated. Three hundred fifty-three surveys were completed, and 120 surveys were partially completed. Three hundred fifty-three completed surveys were included in the results (75%).

### Demographics

The majority of participants indicated that their caseload was > 75% equine (315/353; [89.2%]), 27/353 [7.7%] reported having a caseload between 25 and 75% equine, and the remainder 11/353 [3.1%] reported having a caseload <25% equine. From these answers, 87/353 [24.6%] participant caseload consisted of 75–100% lameness, 113/353 [32%] 50–75% lameness; 103/353 [29.2%] 25–50% lameness, and 50/353 [14.2%] <25% lameness. Participants whose caseload consisted of more than 50% lameness were more likely to use NSIATs compared to practitioners with a lameness caseload <50% (*P* < 0.001).

English sport horses were more commonly treated by participants, followed by recreational riding horses, western performance horses, racehorses, endurance horses, and other disciplines such as gaited, draft, retired, and geriatric horses. English sport horse practitioners were more likely to use NSIATs compared to other disciplines (*P* = 0.02). Participants treating pleasure horses were less likely to use NSIATs (*P* = 0.04) than participants that practiced on other disciplines.

**Figure 1:**
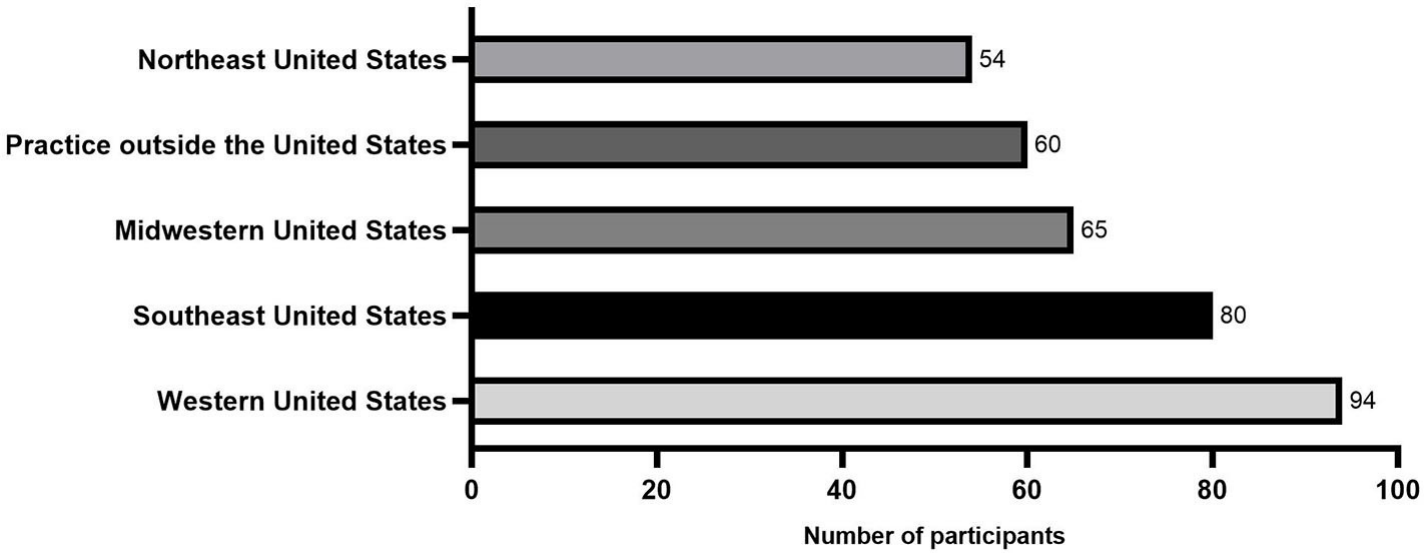
Histogram showing the geographic distribution from a total of 353 equine practitioners that answered the survey.

The majority of survey participants practiced in the USA (293/353[83%]; 80/353 [22.6%] southeastern USA; 54/353 [15.3%] northeast USA.; 65/353 [18.4%] midwestern USA.; and 94/353 [26.6%] western USA). The remainder of participants (60/353 [17%] practiced internationally, including Europe, Canada, Australia, and the Middle East (Figure 1).

The majority of participants had been in practice for >20 years (143/353 [40.5%]), with 96/353 [27.2%] between 10 and 20 years, 75/353 [21.3%] between 5 and 10 years, and 39/353[11.1%] practicing <5 years. The use of NSIATs was not affected by participants' geographic location (USA residents vs. non-USA residents) (*P* = 0.6) or years of experience (*P* = 0.1).

### Injection Frequency

**Figure 2:**
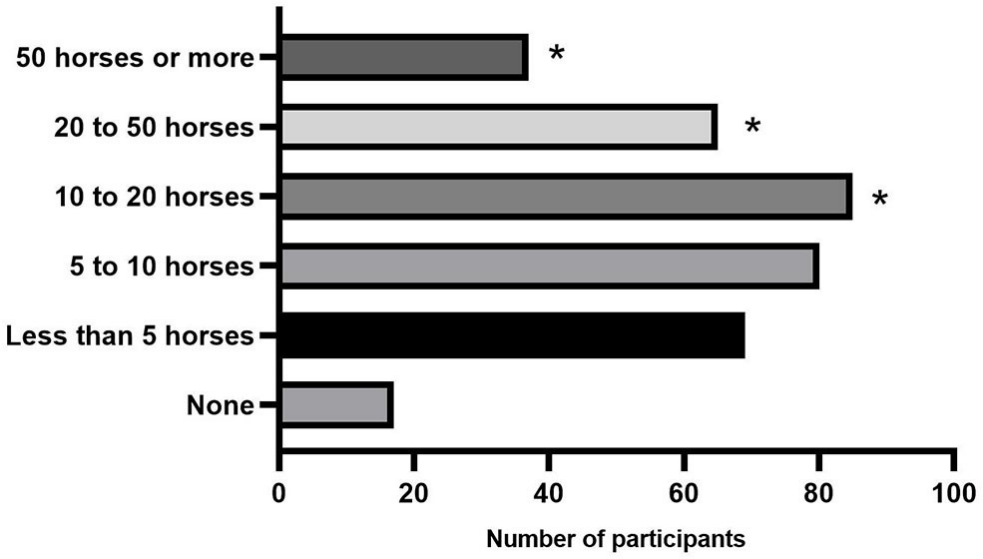
Response count of the number of horses injected per month by participants. * Denotes a significant difference between participants seeing that number of horses that are more likely to use NSAITs (*P* < 0.001).

Participants were asked to estimate the number of horses in which they perform joint injections (steroidal and non-steroidal products) per month (Figure 2). Seventeen/353 [4.8%] did not perform joint injections, 69/353 [19.5%] injected <5 horses/month, 80/353 [22.7%] injected between 5 and 10 horses/month, 85/353 [24.1%] injected between 10 and 20 horses/month, 65/353 [18.4%] injected 20–50 horses/month, and 37/353 [10.5%] injected more than 50 horses in a month. Participants that treated more than 10 horses intra-articularly per month were more likely to use NSIATs (*P* = 0.001).

### Use of NSIATs

Of 336 participants who perform intra-articular injections, 291 used NSIATs in their practice (291/336 [87.5%]), while 42/336 participants [12.5%] did not use these products. Of the participants who did not use NSIATs, 22/42 [52.4%] did not have the product or equipment for processing available in their practice. Twenty/42 [47.6%] did not use NSIATs, no reason was given.

Thirty-eight/291 [12.9%] participants that used NSIATs estimated that they injected <5 horses per year with NSIATs, 39/291 [13.4%] used NSIATs in <10 horses per year, 83/291 [28.5%] used NSIATs in 10–20 patients per year, 66/291 [22.5%] used NSIATs to inject between 20 and 50 horses per year, and 65/291 [22.3%] used NSIATs in more than 50 patients a year.

Participants using NSIATs were asked to rank their top three preferred intra-articular medications, including corticosteroids and hyaluronic acid. The participants were asked to rank at least three products in order of their preferences but were instructed no to rank products that they did not use. The three most popular therapies chosen by the participants were corticosteroids (210/291 [72.2%]), hyaluronic acid (195/291 [67%]), and ACS (90/291 [30.9%]). According to the median value of rank, intra-articular therapies were preferred by participants in the following order (reported as median rank ± interquartile range): Corticosteroids (1 ± 1), hyaluronic acid (2 ± 0), ACS (4 ± 2), APS (4 ± 2), PRP (4 ± 2), cellular therapies (5 ± 2), and polyacrylamide hydrogel (6 ± 3) (Table 2).

**Table 2:** A total of 291 participants ranked NSIATs according to their preferences.

**Rank product**	**1**	**2**	**3**	**4**	**5**	**6**	**7**	**Median**	**Total times product was ranked**
Corticosteroids	210	41	15	3	3	6	20	1	291
Hyaluronic acid	44	195	20	3	8	18	5	2	289
Autologous conditioned plasma (PRP)	4	11	56	75	51	25	15	4	237
Autologous conditioned serum (ACS)	7	8	90	72	46	23	4	4	250
Autologous protein solution (APS)	10	12	76	31	36	28	25	4	218
Cellular therapies	4	16	13	45	48	52	31	5	209
Polyacrylamide hydrogel	12	8	21	28	28	33	68	6	200

*The table indicates the number of practitioners that ranked a product from 1 to 7, the median obtained, and the number of times the product was ranked*.

The three most common reasons for the use of NSIATs were scientific data and articles published regarding product safety and efficacy (105/291 [36.1%]), personal experience with the product (80/291 [27.5%]), and specific conditions being treated (49/291 [16.8%]).

When participants were asked to rank which NSIAT they preferred regardless of client preference or product availability, ACS (96/291 [33%]) and APS (91/291 [31.3%]) were the top choices, followed by PRP (35/291 [12%]), cellular therapies (22/291 [7.6%]), and polyacrylamide gel (17/291 [5.8%]). Thirteen/291 [4.4%] selected other therapies as their first option, indicating a preference to use polyglycan and/or polysulfated glycosaminoglycans (Adequan®). For polyglycan and polysulfated glycosaminoglycans (Adequan®), practitioners did not specify if they were used intra-articularly or systemically. Although this was not specifically asked, in the comment section, participants explained that the main reasons not to choose NSIATs were economic constraints and lack of standardized studies with results regarding product efficacy. Participants with a reduced lameness caseload reported economic difficulties in purchasing equipment to provide NSIATs, preferring to send these cases to a referral institution to be treated with these products.

### Autologous Conditioned Plasma or Platelet-Rich Plasma (PRP)

Survey results for PRP are summarized in Table 3. From the 291 participants that used NSIATs, 225 (77.3%) used PRP, while 66 (22.7%) did not. One hundred and eighty/ 224 [80.4%] participants used commercial kits processed by centrifugation, 18/224 [8%] processed PRP using commercial filtration kits, 17/224 [7.6%] processed PRP by manual centrifugation, and 9/224 [4%] sent out their blood samples to an outside laboratory or referral center to process PRP.

**Table 3:** Summary of each NSIAT included in the survey from a total of 291 participants to the survey.

	**PRP**	**ACS**	**APS**	**Cell Therapy**	**Polyacrylamide hydrogel**
Number of responding practitioners using product for musculoskeletal injuries	224/291; 76.9%	200/291; 68.7%	137/291; 47.1%	142/291; 48.8%	104/291; 35.7%
Number of responding practitioners using product IA	196/291; 67.4%	200/291; 68.7%	137/291; 47.1%	137/291; 47.1%	104/291; 35.7%
Top 2 reasons for practitioner use of the product	1- Ligament/ Tendon pathology (150/224; 66.9%) 2- Acute articular Pathology (25/224; 11.2%)	1-Acute articular Pathology (71/200; 35.5%) 2- Chronic articular pathology (70/200; 35%)	1- Acute articular pathology (54/137; 39.4%) 2- Chronic articular pathology (51/137; 37.2%)	1- Ligament/Tendon Pathology (71/142; 50%) 2- Acute articular pathology (25/142; 17.6%)	1- Chronic Articular Pathology (75/104; 74.3%) 2-Severe OA unresponsive to other treatments (17/104; 16.3%)
Most frequent products used in combination for IA injection	None (161/224; 71.9%)	None (147/200; 73.5%)	None (119/137; 86.9%)	None (79/142; 55.6%)	Not asked

Arthrex ACP® Double Syringe System (69/224 [30.8%] and Restigen PRP® (62/224 [27.7%]) were the commercial kits most commonly used by participants, followed by Harvest® SmartPrep® System (22/224 [9.8%]), E-PET™ Equine Platelet Enhancement Therapy (19/224 [8.5%]), Magellan® Autologous Platelet Separator System (19/224 [8.5%]), and GPS® III Platelet Concentration System (5/224 [2.2%]). Regarding the activation method, 177/224 [79%] of the participants did not activate their PRP before administration, 18/224 [8%] activated platelets with calcium chloride, 9/224 [4%] activated platelets with extracorporeal shockwave therapy, and 9/224 [4%] activated platelets with freeze/thaw cycle(s).

The three most common reasons participants chose PRP were for treatment of ligament or tendon lesions (150/224 [67%]), acute articular pathology 25/224 [11.2%], and chronic articular pathology (23/224 [10.3%]).

One hundred and twenty-one/224 [54%] of participants used systemic anti-inflammatory medication (flunixin meglumine or phenylbutazone) when administering PRP, while 103/224 [46%] did not use any of these medications simultaneously. Ninety-nine/224 [44.2%] participants ensured that the horse was not currently receiving a long-term sedative such as reserpine before collecting and processing PRP, while 125/224 [55.8%] did not ask about this regularly. The majority of participants did not combine PRP with other intra-articular medications or products (161/224 [71.9%]). Twenty-three/224 of participants [10.3%] used antibiotics such as amikacin with PRP, 9/224 [4%] combined PRP with hyaluronic acid, 9/224 [4%] combined PRP with other cellular therapies, and 4/224 [1.8%] combined PRP with corticosteroids. Twenty-eight /224 [12.5%] of participants did not use PRP intra-articularly.

**Figure 3:**
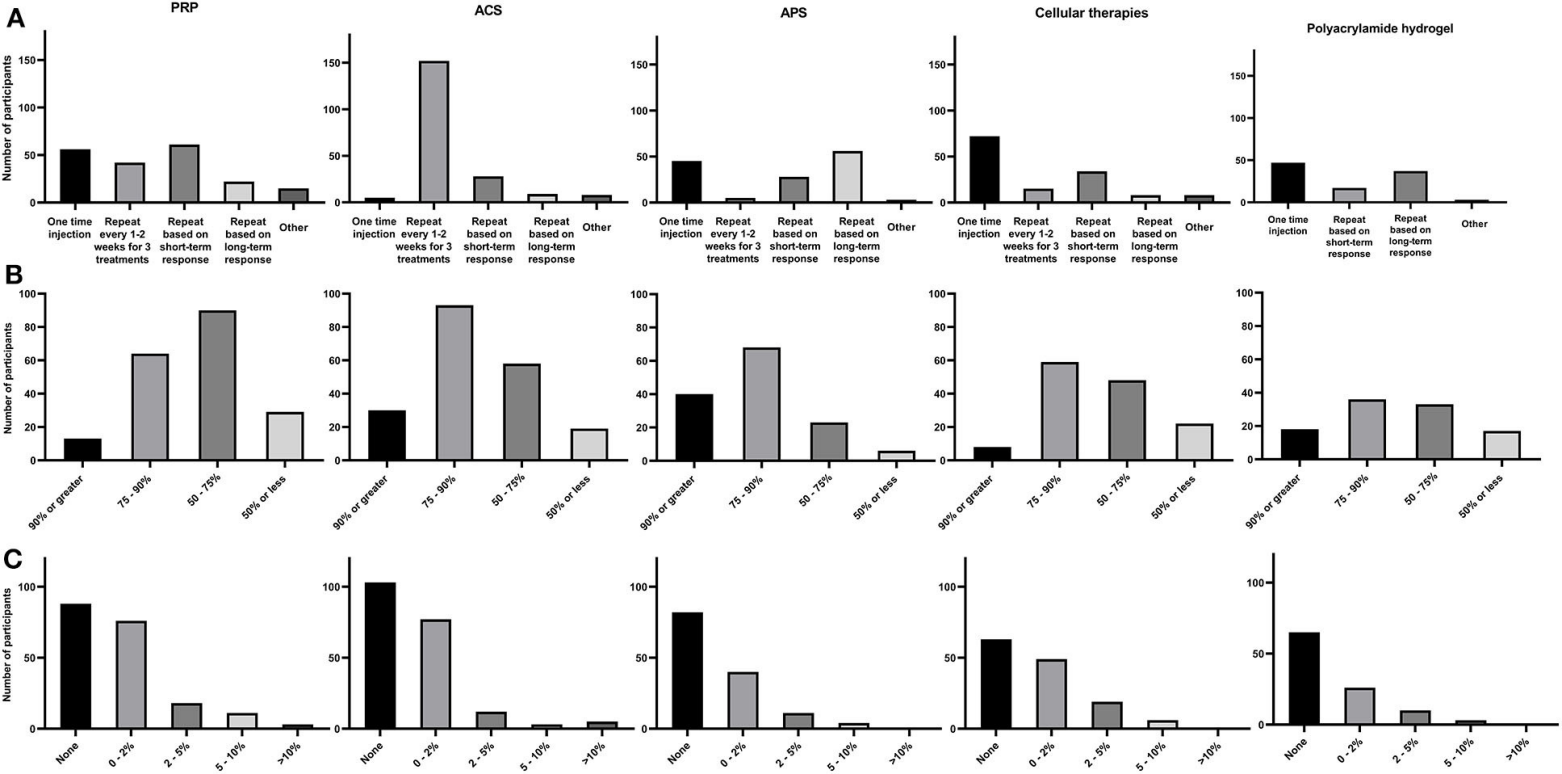
Summary of each NSIAT (columns) included in the survey from a total of 291 participants to the survey. **(A)** Treatment protocol, **(B)** Subjective clinical improvement, and **(C)** Flare rate observed after intra-articular treatment.

Regarding intra-articular treatment protocols, 61/196 [31.1%] of participants repeated injections based on short-term clinical response, 56/196 [28.6%] used PRP as a one-time injection, 42/196 [21.4%] repeated injections every 1–2 weeks for a total of 3 treatments, 22/196 [11.2%] repeated injections based on long term clinical response, and the remainder of participants 15/196 [7.7%] used different personalized protocols (Figure 3A).

When evaluating the subjective assessment of clinical response to PRP, 13/196 [6.6%] estimated that their patients had a 90% or greater improvement, 64/196 [32.7%] participants reported 75–90% improvement in their patients, 90/196 [45.9%] estimated 50–75% improvement in their patients, and 29/196 [14.8%] reported <50% improvement (Figure 3B).

Participants were asked about their impression regarding the incidence of acute joint flares after intra-articular PRP treatment. Eighty-eight of 196 [44.9%] participants reported no incidence of joint flare in their patients, 76/196 [38.8%] participants observed joint flare in <1 horse per 50 injected (2%), 18/196 [9.2%] participants estimated joint flare in <1 horse per 20 horses injected (5%), 11/196 [5.6%] participants estimated joint flare in <1 horse per 10 horses injected (10%), and 3/196 [1.5%] observed joint flare in more than 1 horse per 10 horses injected (Figure 3C).

### Autologous Conditioned Serum (ACS)

Survey results for ACS are summarized in Table 3. From the 291 participants using NSIATs, 200 (68.73%) used ACS, while 91 (31.27%) did not use ACS in their patients. The commercial kits most commonly used by equine participants were the Orthokine® vet IRAP (87/200 [43.5%] and Arthrex-IRAP II™ System (94/200 [47%]), while MediVet ACS was minimally used (5/200 [2.5%]), and the rest of the participants did not specify a kit used (14/200 [7%]).

The three most common reasons for participants to choose ACS were for treatment of acute articular pathology (71/200 [35.5%]), treatment of chronic articular pathology (70/200 [35%]), or for postoperative therapy (28/200 [14%]).

Regarding combinations of ACS with other therapies, 147/200 [73.5%] participants did not combine ACS with any other product; while 25/200 [12.5%] combined ACS with antibiotics like amikacin, 17/200 [8.5%] combined with hyaluronic acid, 7/200 [3.5%] combined with other NSIATs and 4/200 [2%] combined with corticosteroids.

The most common ACS intra-articular treatment protocol was repeated injection every 1–2 weeks for 3 treatments (152/200 [76%]). The next most frequent treatment protocols in descending order were: repeated injections within 3 months based on short-term clinical response (28/200 [14%]), repeated injections within 6 months to a year based on long-term clinical response (9/200 [4.5%]), and one-time injection or various diverse protocols based on disease 11/200 [5.5%] (Figure 3A).

Overall subjective clinical outcome assessment for ACS, 30/200 [15%] participants observed a 90% or more clinical improvement after treatment, 93/200 [46.5%] observed 75–90% improvement in their patients, 58/200 [29%] observed 50–75% improvement, and 18/200 [9.5%] considered that improvement was 50% or less improvement (Figure 3B).

Regarding the incidence of joint flare post-ACS administration, 103/200 [51.5%] reported no observed flare after ACS treatment, 77/200 [38.5%] observed joint flare in 1 per 50 horses injected, 12/200 [6%] observed joint flare in <1 horse per 20 horses injected, 3/200 [1.5%] observed joint flare in <1 horse per 10 horses injected, and 5/200 [2.5%] observed joint flare in more than 1 horse per 10 horses injected (Figure 3C).

### Autologous Protein Solution (APS)

Survey results for APS are summarized in Table 3. A total of 137/291 (47.1%) participants that used NSIATs used APS, while 154/291 (52.9%) did not use APS. During the time the survey was done, Pro-Stride® was the only brand available on the market for processing APS in horses.

The three most common reasons for the use of APS were the treatment of acute articular pathology (54/137 [39.4%]), treatment of chronic articular pathology (51/137 [37.1%]), and for other unspecific disease processes (13/137 [9.5%]).

APS was mainly used alone (119/137 [86.9%]), but 13/137 [9.5%] of participants combined APS with antibiotics like amikacin, and 5/137 [3.6%] combined APS with hyaluronic acid. All the participants that used APS intra-articularly processed one kit for small volume synovial structures such as coffin, fetlock, or tarsometatarsal joint. For large volume synovial structures, such as the stifle, 77/137 [56.2%] processed 1 kit, 52/137 [37.9%] processed 2 kits, 2/137 [1.5%] processed more than 2 kits, and 6/137 [4.4%] indicated that they do not use APS for this purpose.

Eighty-eight/137 [64.2%] participants used systemic anti-inflammatory medications when administering APS, while 49/137 [36.8%] did not. Eighty-four/137 [61.3%] did not check with clients to see if the horse was or had been on long-term sedatives, while 53/137 [38.7%] ensured horses were not receiving these drugs before collecting blood to process APS.

Regarding intra-articular treatment protocols for APS, 56/137 [40.9%] respondents repeated injections within 6 months to a year based on a long-term clinical response, 45/137 [32.8%] used APS as a one-time injection, 28/137 [20.4%] repeated injections within 3 months based on short-term clinical response, and 5/137 [3.7%] used APS as a repeat injection every 1–2 weeks for a total of 3 treatments. The remainder of participants (3/137 [2.2%]) varied the protocol depending on the disease (Figure 3A).

Overall subjective clinical outcome assessment for APS, 40/137 [29.2%] participants observed > 90% improvement, 68/137 [49.6%] observed 75–90% improvement, 23/137 [16.8%] observed 50–75% improvement, and 6/137 [4.4%] observed <50% improvement (Figure 3B).

Eighty-two/137 [59.8%] participants had not observed acute joint flare after treatment with APS, 4/137 [29.2%] observed joint flare in <1 horse per 50 injected, 11/137 [8%] observed joint flare in <1 horse per 20 injected, and 4/137 [3%] observed joint flare in <1 per 10 horses injected (Figure 3C).

### Cellular Therapies

Survey results for cellular therapies are summarized in Table 3. From the 291 participants using NSIATs, 142 (48.8%) used cellular therapies, while 149 (51.2%) did not. One hundred and fifteen/142 [81%] of participants preferred to use autologous cells/tissues, while 24/142 [19%] used allogenic cells/tissues. Eighty-nine/ 142 [62.7%] participants obtained cells from bone marrow, 28/142 [19.7%] from adipose tissue, 21/142 [14.8%] from umbilical cord, 3/142 [2.1%] peripheral blood, and 1/142 [0.7%] from synovial tissue.

The three most common reasons participants used cellular therapies were the treatment of ligament or tendon lesions (71/142 [50%]), treatment of acute articular pathologies (25/142 [17.6%]), and for postoperative treatment (21/142 [14.8%]).

Seventy-nine/142 [55.6%] practitioners did not combine cellular therapies with other products, 26/142 [18.3%] used cellular therapies in combination with hyaluronic acid, 20/142 [14.1%] used cellular therapies with PRP, 6/142 [4.2%] used cellular therapies with ACS, 7/142 [4.9%] used cellular therapies with antibiotics, and 5/142 [3.52%] did not use cellular therapies intra-articularly.

The cellular intra-articular treatment protocol most commonly used by participants was a one-time injection (72/137 [52.6%]). This protocol was followed in frequency by repeated injections based on short-term clinical response (34/137 [24.8%]), repeated injections every 1–2 weeks for a total of 3 treatments (15/137 [11%]), and repeated injections based on the long-term clinical response (8/137 [5.8%]). The remainder of participants (8/137 [5.8%] varied the protocol depending on disease treated (Figure 3A).

Overall subjective clinical outcome assessment of cellular therapies, 8/137 [5.8%] participants observed > 90% improvement, 59/137 [43.1%] observed 75–90% improvement, 48/137 [35.0%] observed a 50–75% improvement, and 22/137 [16.1%] observed <50% improvement (Figure 3B).

Regarding the incidence of joint flare post-administration, 63/137 [46%] reported they had not observed joint flare after treatment with cellular therapies, 49/137 [35.8%] observed joint flare in 1 per 50 horses injected, 19/137 [13.8%] observed joint flare in <1 horse per 20 horses injected, and 6/137 [4.4%] observed joint flare in <1 horse per 10 horses injected. There was no difference between reported flare rate and the use of allogenic vs. autologous cellular therapies (*P* = 0.8) (Figure 3C).

### Polyacrylamide Hydrogel

Survey results for polyacrylamide hydrogel are summarized in Table 3. One hundred and four/291 (35.7%) participants used polyacrylamide hydrogel intra-articularly, while 187 (64.3%) did not. Noltrex™ was the brand most commonly used (61/104 [58.7%]), followed by Vet Arthramid® (38/104 [36.5%]) and VetAquamid® hydrogel reconstruction (5/104 [4.8%]). Participants practicing outside of the USA were more likely to use polyacrylamide hydrogel compared to participants practicing in the USA (*P* < 0.001). Some practitioners practicing in the USA reported difficulties in acquiring this product.

The most common reason for the use of polyacrylamide hydrogel was to treat chronic articular pathologies (75/104 [72.1%]) and severe osteoarthritis unresponsive to other treatments (17/104 [16.3%]).

The most common intra-articular treatment protocols in descending order were a one-time injection (47/104 [45.2%]), repeated injections based on long-term clinical response (37/104 [35.6%]), and repeated injections based on short-term clinical response (17/104 [16.3%]) (Figure 3A).

Regarding subjective clinical outcome assessment of polyacrylamide hydrogel administration, 18/104 [17.3%] participants observed 90% or more improvement, 36/104 [34.6%] observed 75–90% improvement, 33/104 [31.7%] observed 50–75% improvement, and 17/104 [16.4%] observed improvement in <50% of patients (Figure 3B).

Sixty-five/104 [62.5%] reported no acute joint flare after treatment with polyacrylamide hydrogel, 26/104 [25%] observed joint flare in <1 horse per 50 injected, 10/104 [9.6 %] observed joint flare in <1 horse per 20 injected, and 3/104 [2.9%] observed joint flare in <1 per 10 horses injected (Figure 3C).

## Discussion

The results of our study show that equine practitioners and participants frequently use NSIATs, and they were familiar with the different modalities of NSIATs available on the market. Of the 353 practitioners surveyed, 291 (87.5%) use NSIATs. However, when asked which intra-articular therapy they prefer, corticosteroids and hyaluronic acid remained most popular.

Within NSIATs, ACS and PRP were the most commonly used, followed by APS, cellular therapies, and polyacrylamide hydrogel. Practitioners with a higher lameness caseload and those that performed intra-articular injections in more than 10 horses/month were significantly more likely to utilize NSIATs. The most cited reason why practitioners did not use NSIATs was the economic limitations of the client. Commercial kits to process products such as PRP, ACS, or APS often require specific centrifuges, which makes the purchase and use of these products difficult for the veterinarian with a low lameness caseload. Discipline, as previously reported, still influences the use of NSIATs, as English sport horse practitioners were more likely to use NSIATs compared to other disciplines (4).

According to a previous survey, corticosteroids with or without hyaluronic acid were the most common therapies used by members of the American Association of Equine Practitioners (AAEP) (4). In a 2009 survey of equine practitioners, Ferris et al. reported that 54.1% of the participants used ACS intra-articularly when horses were unresponsive to corticosteroid treatment or cost was not an issue for the client. Based on our survey results, it appears that practitioners (68.73%) are using ACS more often than in the past, followed by PRP (67.35%). Additionally, it appears that practitioners are now selecting them to treat acute joint pathology. NSIATs have been on the market and available to participants of the current survey longer than participants of the 2009 survey; this might have increased practitioner and owner awareness as well as willingness to use these products.

In another survey of equine practitioners in 2018, PRP and ACS were considered two of the top 10 rehabilitation modalities for musculoskeletal injuries (12). PRP was used in 98.9% of cases to treat tendon or ligament injuries, while ACS was more frequently used in the joint postoperatively (55.3%) or to maintain performance (32.3%) (12). Similarly, in our survey, PRP and cellular therapies were more frequently used to treat soft tissue injuries (tendons or ligaments), while products such as ACS and APS were used to treat joint disease. Interestingly, cellular therapies were more frequently chosen as intra-articular treatment during the postoperative period compared to other therapies; for example, some practitioners commented about the common use of mesenchymal stem cells (MSCs) in stifle injuries. The stifle is a unique joint in which soft tissue structures (meniscus and ligaments) are contained within the synovial space (13). Use of MSCs for meniscal injuries is likely influenced by a previous publication reporting improved outcomes in horses with stifle injury that were treated with arthroscopic exploration and debridement followed by intra-articular MSCs administration (14).

Cellular products rely on cell-to-cell communication as well as autocrine and paracrine signaling to exert their effects on the tissue's microenvironment in which they are injected. The cellular and molecular mechanisms of MSCs produce their immunomodulatory effect have not been fully clarified yet. A recent publication has shown that MSCs can maintain their anti-inflammatory properties despite being metabolically inactive (15). However, the authors believe that for these products to be effective, cellular viability, as well as function should be maintained. Few studies have evaluated the effects of other therapies on cellular properties *in vitro*. These studies have shown beneficial and deleterious effects on cellular products, and practitioners should be aware of detrimental effects to prevent reduced efficacy and/or death of the cellular product. Most participants in our study (55–85%) did not combine cellular products with other intra-articular medications. When hyaluronic acid was added to MSCs *in vitro*, cellular viability, and chondrogenesis were enhanced (16). However, in another *in vitro* study, no differences in cellular viability or increased production of transforming growth factor-beta was observed when MSCs were cultured with hyaluronic acid (17). Beneficial effects have been observed when combining PRP and MSCS. In an *in vitro* study, PRP enhanced proliferation and chondrogenesis in cultured MSCs (18), and *in vivo*, horses with naturally occurring OA treated intra-articularly with MSCs combined with PRP showed clinical improvement compared to either product alone (8). However, Goodrich et al. reported that the combination of MSCs and PRP enhance bone formation instead of cartilage in osteochondral defects created on the lateral trochlear ridge of the stifle (19). Although no practitioners reported combining corticosteroids with cell therapies, it is important to mention that adding methylprednisolone or triamcinolone to MSC cultures *in vitro* resulted in the rapid death of MSCs (20).

Some practitioners use antibiotics intra-articularly when performing joint injections. Studies investigating the effects of antibiotics (aminoglycosides and fluorinated quinolones) at clinically extrapolated doses added to MSC cultures have shown deleterious effects with marked reduction in cellular viability (17, 21). A recent study that evaluated the effects of clinically relevant doses of antibiotics on chondrocytes *in vitro* resulted in significant cellular death (22). In this survey, 7/142 [4.9%] of participants that used cellular therapies reported using them in combination with antibiotics. Though the effects of these products in combination *in vivo* have not been investigated, a combination of cellular products and antibiotics is not recommended. When using blood or tissue-based products, practitioners should be aware of the positive and negative effects other therapeutics can have on the therapy administered. Approximately 8.5–10.3% of the practitioners combined products like PRP, ACS, and APS with antibiotics. These blood-based products are acellular or have few cells (red and white blood cells). The effect of antibiotics on these products for treatment of inflamed synovial tissues has not been evaluated. The authors recommend caution with the use of antibiotics in combination with NSIATs that are obtained from blood and tissues, particularly if cellular processes are how these products are thought to exert their effects within the synovial environment.

Surveyed practitioners were questioned about their use of NSAIDs when using PRP and APS as these products exert some of their effects through platelet concentration and release of growth factors (10). This question was not asked with the rest of therapies included in the survey (ACS, MSCs, and polyacrylamide hydrogel). In the author's experience, some practitioners elect not to use NSAIDs at the time of PRP administration due to concerns in reducing the inflammatory response within the microenvironment of the diseased tissue, possibly reducing the reparative cellular response that is stimulated with injection of PRP. However, no further studies have been performed to answer this question. On the other hand, few studies have evaluated the simultaneous administration of NSAIDs when preparing blood-derived therapies. PRP obtained from horses receiving ketoprofen achieved higher platelet counts than PRP obtained from horses not receiving ketoprofen (23). Although no growth factors were measured in that study, high platelet counts have been correlated with high concentrations of transforming growth factor-beta, insulin-like growth factor, and platelet-derived growth factor in PRP preparations (24, 25). A recent study evaluated the effects of NSAIDs on platelet aggregation and function. This study found that administration of firocoxib, flunixin meglumine, or phenylbutazone had no effect on platelet aggregation or function (26). Incubation of NSAIDs or corticosteroids with blood prior to processing did not affect concentration of inflammatory (interleukin−1β) or anti-inflammatory proteins (interleukin 1 receptor antagonist protein) in APS (27, 28). However, these were *ex vivo* studies, and results should be confirmed with *in vivo* experiments to evaluate changes in product efficacy. An *in vitro* study where NSAIDs have shown a dose-dependent effect on cultured MSCs (29). A low dose of flunixin meglumine and meloxicam had positive effects on cell proliferation and migration, while a high concentration of these drugs and phenylbutazone produced a significant decrease in cellular viability and proliferation (29). Although the effect of the NSAIDs on some NSIATs is not completely clear *in vivo*, there is no indication that NSAIDs cannot be simultaneously administered with NSIATs.

A recent publication reported that horses receiving reserpine (a long-term sedative), had hypercoagulable blood, especially when attempting to produce autologous biologic products (30). Reserpine produces a detrimental effect, significantly increasing platelet aggregation, thus it is recommended to harvest blood for biological processing before using this medication (30). In our survey, more than 50% of the participants that use PRP and APS did not ask their clients if their horses had received reserpine before blood collection. Considering the effect of this drug on platelet function, equine practitioners should include this question prior to blood collection and processing of blood-based NSIATs, particularly if the horse is on stall rest at the time of collection.

When APS is processed, an average of 3 mL of final product is obtained per kit. In our survey, practitioners were questioned how many kits were used when considering the size of the joint. The majority used one kit for smaller joints and 37.9% used two kits for treatment of larger joints. This is different from what it was used in a clinical study, where horses with naturally occurring OA were treated with two kits per joint independent of the volume of joint being treated (7). Although practitioners have not reported worse outcomes when using a single kit, further investigation to evaluate a possible dose-effect of this drug is warranted.

Our results indicate that practitioners outside the USA more frequently used polyacrylamide hydrogel than practitioners in the USA. Investigations into the use of polyacrylamide hydrogel have been primarily based out of Europe (31, 32), where it has been available on the market for a longer time period than in the USA. These factors indicate that practitioners outside the USA are likely more familiar with the product and have had more opportunities to use this product than practitioners in the USA.

This study has several limitations that warrant further discussion. Data on the number of practitioners that saw the link to the survey posted on social media, received and reviewed the email, and opened the Spur of the Moment newsletter and viewed the link but did not respond is not available, so response rate could not be calculated. Despite the different avenues used to reach as many practitioners as possible, our response rate could be considered low. The reported use of use NSIATs could be higher or lower than the actual use of NSIATs among equine practitioners surveyed in this study. Practitioners that use NSIATs were likely more willing to take time to complete the survey. Investigators ensured that participants were aware that they did not have to use or be familiar with NSIATs to answer the survey. These participants were asked questions on demographics and use of steroidal and NSIATs. Participants were not required to move forward within the survey to answer therapeutic-specific questions if they responded that they did not use NSIATs. Answers to questions regarding clinical response and complication rates were based on estimation and practitioner recall, and these were subjective impressions, not based on clinical records. The authors only questioned participants on the rate of observed joint flare to provide more standardized options across products to the questionnaire; however, the authors recognize that other complications occur with intra-articular injections.

This survey provides information on the clinical use of NSIATs by equine practitioners, illustrating that NSIATs are used routinely to treat joint pathology. However, practitioners still have questions about the efficacy of these products and ideal treatment protocols in horses. Research investigating the disease-modifying effects of these products and investigations into best practices for how and when these products should be used and needed.

## Data Availability Statement

The raw data supporting the conclusions of this article will be made available by the authors, without undue reservation.

## Ethics Statement

The studies involving human participants were reviewed and approved by IRB committee Auburn University. Written informed consent for participation was not required for this study in accordance with the national legislation and the institutional requirements.

## Author Contributions

The survey was conceived and designed by LB and AV. The survey was reviewed by AW, JT, and FC. AV performed the majority of the data acquisition and analysis under the supervision of LB. All authors contributed to data interpretation and manuscript preparation and gave approval for it to be published.

## Conflict of Interest

AB was employed by the company Zoetis. The remaining authors declare that the research was conducted in the absence of any commercial or financial relationships that could be construed as a potential conflict of interest.
