# Supplementary material for: A Survey of Clinical Usage of Non-steroidal Intra-Articular Therapeutics by Equine Practitioners
Source: Front Vet Sci. 2020 Oct 22;7:579967. doi: 10.3389/fvets.2020.579967 (PMC7642446; doi:10.3389/fvets.2020.579967)
Supplement: Supplementary file 1 [file Data_Sheet_1.docx]

**Supplementary item 1. Survey sent electronically to equine practitioners.**

**Clinical usage of non-steroidal intra-articular therapeutics by equine practitioners**

Clinical usage of **non-steroidal intra-articular therapeutics**, including biologic and synthetic products, has increased within recent years. There is limited evidence in the literature regarding clinical usage and outcomes of these products. Non-steroidal intra-articular therapeutics that will be addressed include biologic products such as autologous conditioned serum (i.e. IRAP products such as Orthokine® vet irap 10 or 60, IRAP II System), autologous conditioned plasma (platelet rich plasma, PRP), autologous protein solution (i.e. Pro-strideAPS),  cellular products (i.e. stem/stromal/progenitor cell therapy or concentrated, and tissue particles), and synthetic products including polyacrylamide hydrogel (Noltrex®Vet Synthetic Joint Lubricant, Arthramid®Vet ). The objective of this survey is to establish the current clinical usage of non-steroidal intra-articular medications by equine practitioners.

**Demographics:**

1. Which statement best describes your caseload?
   1. 100% Equine
   2. 75% Equine
   3. 50% Equine
   4. 25% Equine
   5. < 25% Equine
   6. Other (please specify)
2. What is your overall lameness caseload?
   1. 75-100% lameness
   2. 50-75% lameness
   3. 25-50% lameness
   4. 0-25% lameness
3. What is the major disciplinary focus of your practice
   1. Racehorses (Quarter horse, Standardbred, Thoroughbred)
   2. Western Performance (cutting, reining, roping, speed events)
   3. English Sport Horse (Hunter/Jumper, Dressage, Event horses)
   4. Endurance Horse
   5. Recreational Riding Horses
   6. Other (please specify)
4. How long have you been practicing?
   1. Less than 5 years
   2. 5 to 10 years
   3. 10 to 20 years
   4. 20 years or more

**Geographic location question**

5. Where is your primary practice location?

1. Southeast United States
2. Northeast United States
3. Midwestern United States
4. Western United States
5. I practice outside the United States.

If practicing outside of the United States, please enter the country in which you practice.

***Injection Frequency***

How many horses would you estimate that you perform joint injections on within a given month?

- 1. Less than 5 horses
  2. 5 to 10 horses
  3. 10 to 20 horses
  4. 20 to 50 horses
  5. 50 horses or more

1. Do you use non-steroidal intra-articular therapeutics in your practice?
2. Yes
3. No

IF No 🡪 Survey finishes after this question

1. Does your practice have one or more of these products or means to process these products readily available to use?
   1. Yes
   2. No

IF yes, participants will go through the following questions

***Overall Usage of Regenerative Medicine:***

1. On average how many horses do you inject with non-steroidal intra-articular therapeutics per month?
   1. Less than 5 patients
   2. Less than 10 patients
   3. Less than 10-20 patients
   4. More than 20-50 patients
   5. More than 50 patients
2. Please rank the products that you use most commonly in your practice to treat joint related pathology from most used to least used. Click bubbles in order from most used (1) to least used (2-7), leave the products that you do not use blank.

___ Corticosteroids (Triamcinolone, Betamethasone, Methylprednisolone acetate)

___ Hyaluronic Acid

___ Autologous conditioned plasma (Platelet-rich plasma)

___ Autologous conditioned serum (i.e. IRAP products such as Orthokine® vet irap 10 or 60, IRAP II™ System)

___ Autologous protein solution (i.e. ProStride™)

___ Cellular therapy (stem/stromal cells cultured or concentrated, tissue particulates)

___ Polyacrylamide hydrogel (Noltrex™Vet Synthetic Joint Lubricant, Arthramid®Vet)

__ Other (please specify) ____________________________________

1. ​​​Please rank the products that you use most commonly in your practice to treat joint related pathology from most used to least used. Click bubbles in order from most used (1) to least used (2-7), leave the products that you do not use blank.

___Scientific data and articles published regarding the product’s safety and efficacy

___Personal experience with the product

___Availability of the product

___The specific joint being treated

___Cost of product

___Specific condition being treated

___Client Request

­­___ Other: please specify__________________________

1. Please rank at least the top 3  reasons from most influential to least influential to your decision as to which intra-articular therapy you preferentially use. Click bubbles in order from most influential (1) to least influential reason (3-7).

___Autologous conditioned plasma (Platelet rich plasma- PRP)

___Autologous conditioned serum (IRAP products such as Orthokine® vet irap 10 or 60, IRAP II™ System)

___Autologous protein solution (i.e. Prostride™)

___Cellular therapy (stem/stromal cells cultured or concentrated, tissue particulates)

___Polyacrylamide hydrogel (Noltrex™Vet Synthetic Joint Lubricant, Arthramid®Vet)

___ Other (please specify)___________________

***Platelet-Rich Plasma (PRP):***

Autologous conditioned plasma also referred to as platelet-rich plasma (PRP) is a product obtained from the horse’s own blood. The blood is filtered or centrifuged to obtain plasma with an increased number of platelets rich in growth factors.

1. Do you use platelet-rich plasma (PRP) to treat your patients?
   1. Yes
   2. No

If your answer to Question #1 was YES, please answer the questions below.

1. How is the autologous conditioned plasma/platelet rich plasma processed in your practice prior to administration?
   1. Manual centrifugation
   2. Centrifugation using a commercialized kit and centrifuge
   3. Filtration
   4. Other (please specify)_____________
2. If you use a commercialized kit to process PRP, what kit do you use?
   1. Angel® Concentrated Platelet Rich Plasma
   2. Arthrex ACP® Double Syringe System
   3. GPS® III Platelet Concentration System
   4. E-PET™ Equine Platelet Enhancement Therapy
   5. Magellan® Autologous Platelet Separator System
   6. Harvest® SmartPrep® System
   7. Other (please specify): _________________________________________________
3. What activation agent do you use with PRP administration?
   1. None
   2. Bovine thrombin
   3. Calcium chloride
   4. Freeze/thaw cyle(s)
   5. Physiologic stimulation (Allow the wound environment to activate the platelets)
   6. Other (Please explain) _______________________________________
4. Please rank the top 3 most common reasons you use PRP in your patients?

___Preventative/prophylactic measure in performance horse

___Acute articular pathology

___Chronic articular pathology needing “maintenance” or routine injections

___Post-operative therapy

___Ligament or tendon lesions

___Tendon sheath or Bursa applications

___Wound applications

___Laminitis

___Other (please specify):_______________________________

1. When using autologous conditioned plasma/platelet rich plasma, what other non-steroidal intra-articular products do you administer?
   1. None
   2. Antibiotics
   3. Corticosteroids
   4. Cellular therapeutics
   5. Other (please specify):_____________________
2. Do you administer non-steroidal anti-inflammatory medication (Flunixin meglumine or phenylbutazone) when administering PRP?
   1. Yes
   2. No
3. Do you ensure that the horse is not currently on a long-term sedative (i.e. Reserpine) prior to pulling and processing the PRP?
   1. Yes
   2. No
4. What activation agent do you use with PRP administration?
   1. None
   2. Bovine thrombin
   3. Calcium chloride
   4. Freeze/thaw cyle(s)
   5. Physiologic stimulation (Allow the wound environment to activate the platelets)
   6. Other (Please explain) _______________________________________
5. If you use autologous conditioned plasma/platelet rich plasma for intra-articular therapy, what would your typical treatment protocol be?
   1. One time injection
   2. Repeat injection every 1-2 weeks for 3 treatments
   3. Repeat injection based on short-term clinical response (i.e. re-injection performed within 3 months of initial therapy)
   4. Repeat injection based on long-term clinical response (i.e. “Maintenance” therapy performed every 6 mos -1 yr)
   5. Other (please explain) ________________________________________
6. If using autologous conditioned plasma/platelet rich plasma intra-articularly, what would you consider your overall response rate to be?
   1. 90% or greater clinical improvement
   2. 75 to 90% clinical improvement
   3. 50 to 75% clinical improvement
   4. 50% or less clinical improvement
7. When administering autologous conditioned plasma/platelet rich plasma intra-articularly, what incidence of acute joint flares have you encountered post-administration?
   1. None
   2. <1 in 50 horses injected (2%)
   3. < 1 in 20 horses injected (5%)
   4. < 1 in 10 horses injected 10%)
   5. More than 1 flare per 10 horses injected

***Autologous Conditioned Serum (ACS/IRAP):***

Autologous conditioned serum also known as IRAP (Orthokine® vet irap 10 or 60, IRAP II™ System) is obtained from the horses blood following collection into specialized syringes and whole blood incubation. The serum is then collected and administered or aliquots are frozen for subsequent injection.

1. Do you use autologous conditioned serum or IRAP (ACS/IRAP) to treat your patients?
   1. Yes
   2. No

If your answer to Question #1 was YES, please answer the questions below.

1. Which ACS/IRAP product do you commonly use?
   1. Orthokine® vet IRAP
   2. Arthrex-IRAP II™ System
   3. MediVet ACS
   4. Whole blood incubation in medical grade glass container and serum extraction (manual processing)
   5. Other (Please explain) _______________________
2. Please rank the top 3 reasons you will use ACS/IRAP in your patients.

___Preventative/prophylactic measure in performance horse

___Acute articular pathology

___Chronic articular pathology needing “maintenance” or routine injections

___Post-operative therapy

___Ligament or tendon lesions

___Tendon sheath or Bursa applications

___Wound applications

___Other (please explain)____________________________________

1. When using ACS/IRAP intra-articularly, what additional products do you administer with ACS/IRAP?
   1. None
   2. Antibiotics (i.e. amikacin, gentamicin)
   3. Corticosteroids
   4. Hyaluronic acid
   5. Autologous protein solution (i.e. ProStride™)
   6. Autologous conditioned plasma or platelet rich plasma (PRP)
   7. Cellular therapy (stem/stromal/progenitor cell therapy cultured or concentrated, tissue particles)
   8. Other (please specify) _____________________________________
2. If you use ACS/IRAP intra-articularly, what would your typical treatment protocol be?
   1. One time injection
   2. Repeat injection every 1-2 weeks for 3 treatments
   3. Repeat injection based on short-term clinical response (i.e. re-injection performed within 3 months of initial therapy)
   4. Repeat injection based on long-term clinical response (i.e. “Maintenance” therapy performed every 6 mos -1 yr)
   5. Other (please explain) _______________________
3. If using ACS/IRAP intra-articularly, what would you consider your overall response rate to be?
   1. 90% or greater clinical improvement
   2. 75 to 90% clinical improvement
   3. 50 to 75% clinical improvement
   4. 50% or less clinical improvement
4. When administering ACS intra-articularly, what incidence of acute joint flares have you encountered post-administration?
   1. None
   2. <1 in 50 horses injected (2%)
   3. < 1 in 20 horses injected (5%)
   4. < 1 in 10 horses injected 10%)
   5. More than 1 flare per 10 horses injected

***Autologous Protein Solution (APS- Pro-stride™):***

Autologous protein solution (i.e. Pro-Stride^TM^ APS) is an autologous product obtained from the horse’s blood. The blood is first processed using a kit and centrifugation to obtain plasma with concentrated platelets. This plasma is then harvested and processed in a kit that allows exposure of the cellular components of the plasma to polyacrylamide beads enhancing their production of anti-inflammatory proteins during a second centrifugation cycle.

1. Do you use autologous protein solution (APS, Pro-Stride^TM^) in your patients?
   1. Yes
   2. No

If your answer to Question #1 was YES, please answer the questions below.

1. Please rank the top 3 reasons you will use APS (Pro-Stride^TM^ ) in your patients.
   1. ___Preventative/prophylactic measure in performance horse
   2. ___Acute articular pathology
   3. ___Chronic articular pathology needing “maintenance” or routine injections
   4. ___Post-operative therapy
   5. ___Ligament or tendon lesions
   6. ___Tendon sheath or Bursa applications
   7. ___Wound applications
   8. ___Other (please explain):_____________________________________
2. What additional products do you administer intra-articularly with APS (Pro-Stride^TM^) ?
   1. None
   2. Antibiotics (i.e. amikacin, gentamicin)
   3. Corticosteroids
   4. Hyaluronic acid
   5. Cellular therapeutics (stem/stromal or progenitor cell therapy-cultured or concentrated)
   6. Other (please explain):__________________________
3. How many APS (Pro-Stride^TM^ ) kits do you process and administer for large volume synovial structures (i.e. stifle)?
   1. 1 kit
   2. 2 kits
   3. >2 kits
   4. I do not use APS for this purpose
4. How many APS (Pro-Stride^TM^) kits do you use for small volume synovial structures (i.e. coffin, fetlock, etc joints)?
   1. 1 kit
   2. 2 kits
   3. >2 kits
   4. I do not use APS for this purpose
5. Do you administer systemic non-steroidal anti-inflammatory medication when administering APS (Pro-Stride^TM^)?
   1. Yes
   2. No
6. Do you ensure that the horse is not currently on a long-term sedative (i.e. Reserpine) prior to pulling and processing the APS (Pro-Stride^TM^)?
   1. Yes
   2. No
7. If using APS (Pro-Stride^TM^ ) intra-articularly, what would you consider your overall response rate to be?
   1. 90% or greater clinical improvement
   2. 75 to 90% clinical improvement
   3. 50 to 75% clinical improvement
   4. 50% or less clinical improvement
8. If you use APS (Pro-Stride^TM^ ) for intra-articular therapy, what would your treatment protocol be?
   1. One time injection
   2. Repeat injection every 1-2 weeks for 3 treatments
   3. Repeat injection based on short-term clinical response (i.e. re-injection performed within 3 months of initial therapy)
   4. Repeat injection based on long-term clinical response (i.e. “Maintenance” therapy performed every 6 mos -1 yr)
   5. Other (please explain) _______________________
9. When administering APS (Pro-Stride^TM^ ) intra-articularly, what incidence of acute joint flares have you encountered post-administration?
   1. None
   2. <1 in 50 horses injected (2%)
   3. < 1 in 20 horses injected (5%)
   4. < 1 in 10 horses injected 10%)
   5. More than 1 flare per 10 horses injected

***Cellular therapeutics:***Cellular therapeutics would include the following products:

- Cells (stem/stromal and/or progenitor) contained within **tissue particles** (i.e. Pulpcyte® Vet Graft). These products are typically shipped directly from the company.
- Progenitor and stem/stromal cell **concentrates**. These products are obtained after harvesting tissue (adipose or bone marrow) and concentration of the cells from the tissue via centrifugation with or without prior tissue digestion (i.e. Adipose derived stromal vascular fraction or bone marrow aspirate concentrate).
- **Cultured** cellular therapy. These products are obtained after harvesting tissue (adipose, bone marrow, blood, etc) and sending the tissues to a commercial laboratory for culture. The cultured cells would then be shipped back to the practitioner for injection at least 2 weeks or more after the tissue harvest.

1. Do you use cellular therapeutics in your patients?
   1. Yes
   2. No

If your answer to Question #1 was YES, please answer the questions below.

1. Please rank the top 3 most common reasons you use cellular therapy in your patients?
   1. ___Preventative/prophylactic measure in performance horse
   2. ___Acute articular pathology
   3. ___Chronic articular pathology needing “maintenance” or routine injections
   4. ___Post-operative therapy
   5. ___Ligament or tendon lesions
   6. ___Tendon sheath or Bursa applications
   7. ___Wound applications
   8. ___Laminitis treatment
   9. ___Other:__________________________________
2. When using cellular therapy, what is the most tissue source that you most commonly harvest from?
   1. Bone marrow
   2. Adipose
   3. Peripheral Blood
   4. Synovial tissues
   5. Other (please specify)______________________
3. When using cellular therapy, what is the donor source for the cellular therapeutic that you are most commonly using?
   1. Autologous (Obtained from the same horse that the product is to be used)
   2. Allogeneic (Obtained from a different horse than the horse that the product will be injected into)
   3. Xenogeneic (Obtained from a different species)
4. What additional products, if any, do you administer intra-articularly with stem cells?
5. None
6. Antibiotics (i.e. amikacin, gentamicin)
7. Corticosteroids
8. Hyaluronic acid
9. Autologous conditioned plasma (PRP)
10. Autologous conditioned serum (IRAP)
11. Autologous protein solution (APS)
12. Other (please explain):_________________________________
13. If using cellular therapy intra-articularly, what would you consider your overall response rate to be?
14. 90% or greater clinical improvement
15. 75 to 90% clinical improvement
16. 50 to 75% clinical improvement
17. 50% or less clinical improvement
18. If you use cellular therapy for intra-articular therapy, what would your treatment protocol be?
    1. One time injection
    2. Repeat injection every 1-2 weeks for 3 treatments
    3. Repeat injection based on short-term clinical response (i.e. re-injection performed within 3 months of initial therapy)
    4. Repeat injection based on long-term clinical response (i.e. “Maintenance” therapy performed every 6 mos -1 yr)
    5. Other (please explain) _______________________
19. When administering cellular therapy intra-articularly, what incidence of acute joint flares have you encountered post-administration?
    1. None
    2. <1 in 50 horses injected (2%)
    3. < 1 in 20 horses injected (5%)
    4. < 1 in 10 horses injected 10%)
    5. More than 1 flare per 10 horses injected

***Polyacrylamide Hydrogel (Noltrex™ Vet, Arthramid®Vet, Aquamid®, etc)***

Polyacrylamide Hydrogel (PAHG, Noltrex™ Vet, Arthramid®Vet, Aquamid®, etc) is a synthetic product injected intra-articularly. It is incorporated into the synovial lining and provides enhanced viscoelasticity to the synovial fluid.

1. Do you use polyacrylamide hydrogel on your patients?
   1. Yes
   2. No

If your answer to Question #1 was YES, please answer the questions below.

1. Which Polyacrylamide hydrogel product do you most commonly use?
   1. Noltrex™ Vet
   2. Arthramid® Vet
   3. Aquamid® hydrogel reconstruction
   4. Other (Please explain) _______________________
2. Please rank at least the top 3 reasons you will use  polyacrylamide hydrogel in your patients. Click bubbles in order from most important (1) to least (3-5) important reason.
   1. Preventative/prophylactic measure in performance horse
   2. Acute articular pathology
   3. Chronic articular pathology needing “maintenance” or routine injections
   4. Post-operative therapy

Other (please explain) :_________________________________

1. If using polyacrylamide hydrogel intra-articularly, what would you consider your overall response rate to be?
   1. 90% or greater clinical improvement
   2. 75 to 90% clinical improvement
   3. 50 to 75% clinical improvement
   4. 50% or less clinical improvement
2. If you use polyacrylamide hydrogel for treatment of a synovial structure, what would your treatment protocol be?
   1. Once
   2. Repeat injection based on short-term clinical response (i.e. re-injection performed within 3 months of initial therapy)
   3. Repeat injection based on long-term clinical response (i.e. “Maintenance” therapy performed every 6 mos -1 yr)
3. When administering PAAG intra-articularly, what incidence of acute joint flares have you encountered post-administration?
   1. None
   2. <1 in 50 horses injected (2%)
   3. < 1 in 20 horses injected (5%)
   4. < 1 in 10 horses injected 10%)
   5. More than 1 flare per 10 horses injected

End of the survey 🡪 Please add any questions or comments regarding this survey.
